# Economic Burden of Human Papillomavirus-Related Diseases in Italy

**DOI:** 10.1371/journal.pone.0049699

**Published:** 2012-11-21

**Authors:** Gianluca Baio, Alessandro Capone, Andrea Marcellusi, Francesco Saverio Mennini, Giampiero Favato

**Affiliations:** 1 Department of Statistical Science, University College London, London, United Kingdom; 2 Biostatistics Unit, Department of Statistics, University of Milano–Bicocca, Milan, Italy; 3 Institute of Leadership and Management in Health (ILMH), Kingston University London, London, United Kingdom; 4 CEIS Sanità (CHEM – Centre for Health Economics and Management), Faculty of Economics, University of Tor Vergata, Rome, Italy; Universidad Nacional de La Plata, Argentina

## Abstract

**Introduction:**

Human papilloma virus (HPV) genotypes 6, 11, 16, and 18 impose a substantial burden of direct costs on the Italian National Health Service that has never been quantified fully. The main objective of the present study was to address this gap: (1) by estimating the total direct medical costs associated with nine major HPV-related diseases, namely invasive cervical cancer, cervical dysplasia, cancer of the vulva, vagina, anus, penis, and head and neck, anogenital warts, and recurrent respiratory papillomatosis, and (2) by providing an aggregate measure of the total economic burden attributable to HPV 6, 11, 16, and 18 infection.

**Methods:**

For each of the nine conditions, we used available Italian secondary data to estimate the lifetime cost per case, the number of incident cases of each disease, the total economic burden, and the relative prevalence of HPV types 6, 11, 16, and 18, in order to estimate the aggregate fraction of the total economic burden attributable to HPV infection.

**Results:**

The total direct costs (expressed in 2011 Euro) associated with the annual incident cases of the nine HPV-related conditions included in the analysis were estimated to be €528.6 million, with a plausible range of €480.1–686.2 million. The fraction attributable to HPV 6, 11, 16, and 18 was €291.0 (range €274.5–315.7 million), accounting for approximately 55% of the total annual burden of HPV-related disease in Italy.

**Conclusions:**

The results provided a plausible estimate of the significant economic burden imposed by the most prevalent HPV-related diseases on the Italian welfare system. The fraction of the total direct lifetime costs attributable to HPV 6, 11, 16, and 18 infections, and the economic burden of noncervical HPV-related diseases carried by men, were found to be cost drivers relevant to the making of informed decisions about future investments in programmes of HPV prevention.

## Introduction

Although infection by the human papillomavirus (HPV) is often asymptomatic and self-limiting, it has been established to be the cause of a number of clinically significant conditions [1]. The high-risk oncogenic variants of HPV, specifically genotypes 16 and 18, account for approximately 70% of all cases of invasive cervical cancer and cervical dysplasia worldwide [2], [3], as well as a lesser proportion of cases of cancer of the vulva, vagina, anus, penis, or head and neck [4]. The low-risk HPV variants of genotypes 6 and 11 are involved mostly in the aetiopathogenesis of benign external anogenital warts [5], [6] and of virtually all cases of recurrent respiratory papillomatosis (RRP) [7], [8]. Collectively, HPV 6, 11, 16, and 18 impose an important burden of direct costs on the Italian National Health Service, which has not previously been fully quantified.

Primary prevention of HPV-related diseases may be achieved by vaccination. Two preventive vaccines against cervical cancer are currently approved in Italy: 1) a quadrivalent vaccine that protects against HPV types 6, 11, 16, and 18 (Gardasil®) and 2) a bivalent vaccine that protects against HPV types 16 and 18 (Cervarix®). In March 2008, a national HPV immunisation programme that uses the quadrivalent vaccine and targets eleven-year-old girls was initiated, This policy decision was informed by several Italian economic studies confirming the cost-effectiveness of the anti-HPV vaccination for the prevention of cervical cancer, cervical dysplasia, and anogenital warts [9]–[15]. There are currently no published studies that estimate the direct medical costs attributable to noncervical conditions or assess the total economic burden of HPV-related diseases affecting both men and women in Italy.

**Figure 1:**
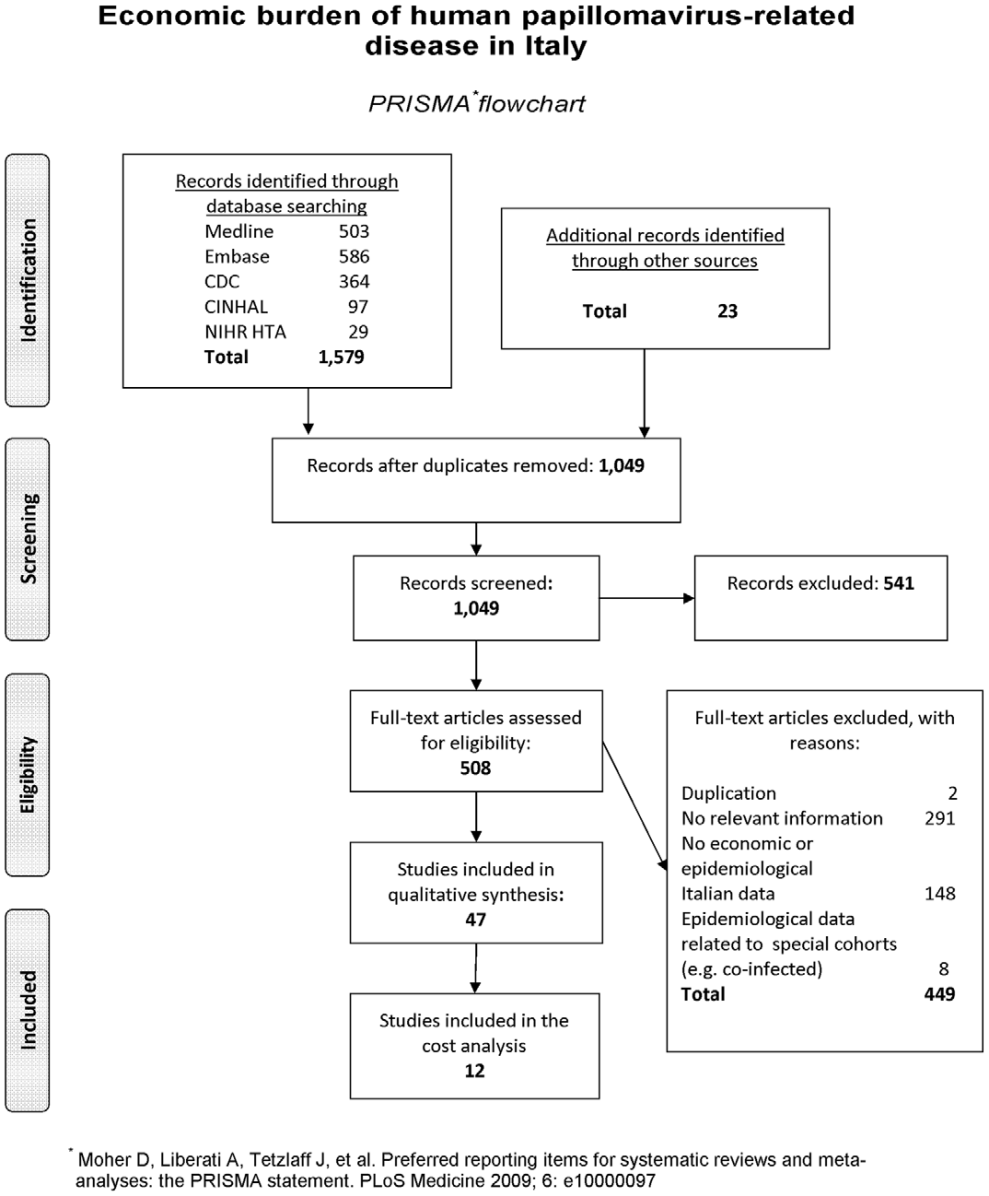
Economic burden of human papillomavirus-related diseases in Italy. Flow of information through the different phases of the systematic review.

The main objective of the study reported herein was to address the foregoing gap: (1) by estimating the total direct medical costs associated with nine major HPV-related diseases, namely invasive cervical cancer, cervical dysplasia, cancer of the vulva, vagina, anus, penis, and head and neck, anogenital warts, and RRP and (2) by providing an aggregate measure of the total economic burden attributable to infection with HPV 6, 11, 16, or 18. The latter value could be useful to inform further studies of cost-effectiveness and analyses of budget impact, and ultimately future public health policy decisions in Italy.

**Table 1:** estimates of lifetime cost per incident patient by diagnosis.

*Conditions*	*Surgical procedures and medical treatments*	*Direct cost per procedure or medical treatment*	*Compound Consumer Price Index rate ^Ŧ^*	*Lifetime direct costs per incident patient (2011 Euro)*	*References*
		a	B	a × b	
***Cervical cancer***	Management of primary tumor	€ 13,122	110.60%	€ 14,513	[11]
	Discounted cost of progression/recurrence after 1 year	€ 8,827	110.60%	€ 9,763	[11]
	Lifetime cost per patient	€ 21,950	110.60%	€ 24,276	
***Cervical dysplasia***	Repeated Pap smears	€ 15	110.60%	€ 17	[15]
	Management of abnormal Pap smears	€ 250	110.60%	€ 277	[15]
	Diagnosis of cervical dysplasia	€ 34	112.92%	€ 38	[12]
	CIN 1 treatment	€ 262	112.92%	€ 296	[12]
	CIN2 treatment	€ 1,439	112.92%	€ 1,625	[12]
	CIN3 treatment	€ 2,011	112.92%	€ 2,271	[12]
***Anal cancer*** †	Diagnosis of malignancy	€ 1,421	110.60%	€ 1,572	DRG 411 [22]
	Anal surgery	€ 3,245	110.60%	€ 3,589	DRG 157 [22]
	Radiotherapy	€ 3,096	110.60%	€ 3,424	DRG 409 [22]
	Minor reconstructive surgery	€ 2,460	110.60%	€ 2,720	DRG 266 [22]
	Follow-up clinical examination (×2)	€ 21	110.60%	€ 46	Outpatient tariff 89.7 [23]
	Follow-up 2-sector CT scan (×2)	€ 88	110.60%	€ 391	Outpatient tariff 88.01.3 [23]
	Lifetime cost per patient			€ 11,742	
***Head and neck cancer*** †	Diagnosis of head and neck malignancy	€ 3,292	110.60%	€ 3,641	DRG 64 [22]
	Head and neck surgery	€ 7,512	110.60%	€ 8,308	DRG 49 [22]
	Radiotherapy	€ 3,096	110.60%	€ 3,424	DRG 409 [22]
	Minor reconstructive surgery	€ 2,460	110.60%	€ 2,720	DRG 266 [22]
	Follow-up clinical examination (×2)	€ 21	110.60%	€ 46	Outpatient tariff 89.7 [23]
	Follow-up 2-sector CT scan (×2)	€ 83	110.60%	€ 368	Outpatient tariff 88.01.3 [23]
	Lifetime cost per patient			€ 18,507	
***Vulvar cancer*** ††	Diagnosis of malignancy	€ 1,421	110.60%	€ 1,572	DRG 411 [26]
	Minor vulvectomy	€ 7,141	110.60%	€ 7,898	DRG 353 [26]
	Radiotherapy	€ 3,096	110.60%	€ 3,424	DRG 409 [26]
	Follow-up clinical examination (×2)	€ 21	110.60%	€ 46	Outpatient tariff 89.26 [27]
	Follow-up 2-sector CT scan (×2)	€ 88	110.60%	€ 391	Outpatient tariff 88.01.3 [27]
	Lifetime cost per patient			€ 13,330	
***Vaginal cancer*** ††	Diagnosis of malignancy	€ 1,421	110.60%	€ 1,572	DRG 411 [22]
	Vaginal surgery	€ 6,736	110.60%	€ 7,450	DRG 354 [22]
	Minor reconstructive surgery	€ 2,734	110.60%	€ 3,024	DRG 356 [22]
	Radiotherapy	€ 3,096	110.60%	€ 3,424	DRG 409 [22]
	Follow-up clinical examination (×2)	€ 21	110.60%	€ 46	Outpatient tariff 89.26 [23]
	Follow-up 2-sector CT scan (×2)	€ 88	110.60%	€ 391	Outpatient tariff 88.01.3 [23]
	Lifetime cost per patient			€ 15,906	
***Penile cancer*** †††	Diagnosis of malignancy	€ 1,421	110.60%	€ 1,572	DRG 411 [22]
	Surgery	€ 4,173	110.60%	€ 4,616	DRG 344 [22]
	Radiotherapy	€ 3,096	110.60%	€ 3,424	DRG 409 [22]
	Follow-up clinical examination (×2)	€ 21	110.60%	€ 46	Outpatient tariff 89.7 [23]
	Follow-up 2-sector CT scan (×2)	€ 88	110.60%	€ 391	Outpatient tariff 88.01.3 [23]
	Lifetime cost per patient			€ 10,048	
***Anogenital warts (women)***	Direct outpatient costs for new patients	€ 368	112.92%	€ 416	[19]
	Discounted cost of recurrence after 1 year (50.6% prob.)	€ 176	112.92%	€ 199	[19]
	Discounted cost of resistance after 1 year (28.2% prob.)	€ 43	112.92%	€ 48	[19]
	Lifetime cost per female patient	€ 587	112.92%	€ 663	[19]
***Anogenital warts (men)***	Direct outpatient costs for new patients	€ 275	112.92%	€ 311	[19]
	Discounted cost of recurrence after 1 year (34.8% prob.)	€ 59	112.92%	€ 66	[19]
	Discounted cost of resistance after 1 year (38.5% prob.)	€ 82	112.92%	€ 92	[19]
	Lifetime cost per male patient	€ 416	112.92%	€ 470	
***RRP*** ‡, ††††	Management of complicated throat infection	€ 5,930	110.60%	€ 6,559	DRG 79 [22]
	Acute clinical treatment of throat infection (×3)	€ 7,006	110.60%	€ 23,246	DRG 76 [22]
	Minor throat surgery (×4.4)	€ 3,263	110.60%	€ 15,878	DRG 63 [22]
	Tracheostomy (11% rate)	€ 9,040	110.60%	€ 1,100	DRG 75 [22]
	Annual cost per patient			€ 46,783	
	Lifetime cost per patient			€ 187,428	

Ŧ. Costs have been adjusted for inflation at 2011 values using compound annual Italian National Consumer Price Indexes (ISTAT reference) from 2005 (112.92%) or 2006 (110.6%).

† . Surgical procedures and medical treatments were modelled on the basis of the Italian AIOM guidelines [16].

†† . Surgical procedures and medical treatments were modelled on the basis of the Italian SIGO guidelines [17].

††† . Surgical procedures and medical treatments were modelled on the basis of the Italian AIRC guidelines [18.].

‡ Respiratory recurrent papillomatosis (RRP).

†††† . Due to the lack of Italian data on RRP, surgical procedures and medical treatments were informed on the basis of U.S. data [20], [21].

## Methods

### Study design

For each of the nine HPV induced diseases we performed an extensive review of the literature to identify the best secondary data available to produce lifetime cost per case estimates, which were expressed in present value. Using an incidence-based approach, we then used the estimated costs per case to produce an aggregate measure of economic burden. The relative prevalence of HPV types 6, 11, 16, and 18 was then applied to the economic burden for each condition to estimate the aggregate fraction of the total burden attributable to HPV infection.

**Table 2:** Estimates of total lifetime cost per case.

	*Conditions*	*Lifetime direct costs per incident patient (2012 Euro)*	*Estimated incident cases per year*	*Lower bound*	*Upper bound*	*Total lifetime direct costs per diagnosis (mill Euro)*	*Lower bound*	*Upper bound*	*References*
		a	b	C	d	a × b	a × c	a × d	
*e*	Cervical cancer	€ 24,276	3,029	2,754	3,304	€ 73.5	€ 66.9	€ 80.2	[26]
*f*	Repeated Pap smears	€ 17	415,000	-	-	€ 6.9	€ 6.9	€ 6.9	[15]
*g*	Management of abnormal Pap smears	€ 277	116,000	-	-	€ 32.1	€ 32.1	€ 32.1	[15]
									
*h*	Diagnosis of cervical dysplasia	€ 38	390,000	-	-	€ 14.9	€ 14.9	€ 14.9	[12]
*i*	CIN 1 treatment	€ 296	21,308	-	-	€ 6.3	€ 6.3	€ 6.3	[12]
*l*	CIN2 treatment	€ 1,625	3,218	3,218	7,638	€ 5.2	€ 5.2	€ 12.4	[12], [34]
*m*	CIN3 treatment	€ 2,271	3,518	3,518	8,350	€ 8.0	€ 8.0	€ 19.0	[12], [34]
*n*	Total cervical lesions (h + i + l + m)					€ 34.4	€ 34.4	€ 52.6	
*o*	Total cervical dysplasia (f + g + n)					€ 73.4	€ 73.4	€ 91.5	
*p*	**Total cervical conditions (e + o)**					**€ 146.9**	**€ 140.2**	**€ 171.7**	
*q*	Anal cancer	€ 11,742	889	704	1,075	€ 10.4	€ 8.3	€ 12.6	[26]
*r*	Head and neck cancer	€ 18,507	12,106	10,140	14,073	€ 224.0	€ 187.7	€ 260.5	[26]
*s*	Vulvar cancer	€ 13,330	1,134	983	1,284	€ 15.1	€ 13.1	€ 17.1	[26]
*t*	Vaginal cancer	€ 15,906	251	207	295	€ 4.0	€ 3.3	€ 4.7	[26]
*u*	Penile cancer	€ 10,048	380	324	435	€ 3.8	€ 3.3	€ 4.4	[26]
*v*	Total anogenital warts (women)	€ 663	62,250	-	-	€ 41.3	€ 41.3	€ 41.3	[13], [19], [27]
*z*	Total anogenital warts (men)	€ 470	80,000	-	-	€ 37.6	€ 37.6	€ 37.6	[13], [19], [27]
*y*	RRP ‡	€ 187,428	243	243	728	€ 45.5	€ 45.5	€ 136.4	[7], [8]
*w*	**Total non-cervical conditions (q + r + s + t + u + v + z + y)**					**€ 381.7**	**€ 339.9**	**€ 514.4**	
	**Total burden (p + w)**					**€ 528.6**	**€ 480.1**	**€ 686.2**	

‡ Respiratory recurrent papillomatosis (RRP).

### Literature review

As mentioned above, the secondary data used to inform estimates were identified via a literature review. The flow of information through the different phases of the systematic review is shown in Figure 1.

**Table 3:** Prevalence of HPV 6, 11, 16, 18.

*Condition*	*Mean prevalence rates chosen to inform the cost model*	*Lower bound*	*Upper bound*	*References*	*Range of prevalence rates identified from the literature review*		*References*
					Lowest	Highest	
Cervical cancer	72.1%	64.9%	84.0%	[26]	70%	77%	[30]-[33]
CIN1	38.1%	32.2%	45.3%	[26]	-	-	-
CIN2	57.2%	46.7%	71.4%	[26]	60.80%	>80%	[35], [36], [37]
CIN3	57.2%	46.7%	71.4%	[26]	77.60%	> 80%	[35], [36], [37]
Anal cancer	64.3%	35.1%	87.7%	[26]	72%	>80%	[39]–[46]
Vulvar Cancer	41.1%	28.1%	55.0%	[26]	40%	57%	[45]–[51]
Vaginal cancer	66.9%	-	-	[26]	40%	69.90%	[45], [46], [51]–[52]
Head and neck cancer	26.4%	24.7%	27.2%	[26]	23.50%	43.50%	[53]–[55]
Penile cancer	51.9%	37.6%	66.0%	[26]	46.30%	79.80%	[56]–[58]
Anogenital warts (man and women)	90%	-	-	[19]	88%	100%	[39], [59]–[60]
RRP ‡	85%	85%	100%	[7], [8]	85%	100%	[59], [61]–[62]

‡ Respiratory recurrent papillomatosis (RRP).

#### Search strategy and selection criteria

In order to collect relevant secondary data, we carried out a systematic search of the following electronic databases: MEDLINE (PubMed), EMBASE (accessed through OVID SP), CDC, CINHAL, and NIHR HTA, covering the period 1990–2011. Details of the search strategy can be found in the Supporting Information files, Appendix S1.

**Table 4:** HPV 6, 11, 16, 18- attributable fraction of total lifetime costs per diagnosis.

	*Conditions*	*Total lifetime direct costs per disease (mill Euro)*	*HPV 16,18 fraction*	*HPV 6,11 fraction*	*HPV 6,11,16,18 fraction*	*Lower bound*	*Upper bound*	*HPV 6,11,16,18 attributable costs (mill Euro)*	*Lower bound*	*Upper bound*	*References*
		a			b	C	d	a × b	a × c	a × d	
*e*	Cervical cancer	€ 73.5	72.1%	0.0%	72.1%	64.9%	84.0%	€ 53.0	€ 47.7	€ 61.8	[26]
*f*	Repeated Pap smears	€ 6.9	80.0%	11.5%	91.5%	-	-	€ 6.3	€ 6.3	€ 6.3	[15]
*g*	Management of abnormal Pap smears	€ 32.1	85.0%	9.0%	94.0%	-	-	€ 30.1	€ 30.1	€ 30.1	[15]
*h*	Diagnosis of cervical dysplasia	€ 14.9	-	-	35.0%	-	-	€ 5.2	€ 5.2	€ 5.2	[28]
*i*	CIN 1 treatment	€ 6.3	27.6%	10.5%	38.1%	32.2%	45.3%	€ 2.4	€ 2.0	€ 2.9	[26]
*l*	CIN2 treatment	€ 5.2	55.7%	1.5%	57.2%	46.7%	71.4%	€ 3.0	€ 2.4	€ 3.7	[26]
*m*	CIN3 treatment	€ 8.0	55.7%	1.5%	57.2%	46.7%	71.4%	€ 4.6	€ 3.7	€ 5.7	[26]
*n*	Total cervical lesions (h + i + l + m)							€ 15.2	€ 13.4	€ 17.5	
*o*	Total cervical dysplasia (f + g + n)							€ 51.6	€ 49.9	€ 53.9	
*p*	**Total cervical conditions (e + o)**	**€ 146.9**						**€ 104.6**	**€ 97.6**	**€ 115.7**	
*q*	Anal cancer	€ 10.4	-	-	64.3%	35.1%	87.7%	€ 6.7	€ 3.7	€ 9.2	[26]
*r*	Head and neck cancer	€ 224.0	-	-	26.4%	24.7%	27.2%	€ 59.1	€ 55.3	€ 60.9	[26]
*s*	Vulvar cancer	€ 15.1	-	-	41.1%	28.1%	55.0%	€ 6.2	€ 4.2	€ 8.3	[26]
*t*	Vaginal cancer	€ 4.0	57.6%	9.30%	66.9%	-	-	€ 2.7	€ 2.7	€ 2.7	[26]
*u*	Penile cancer	€ 3.8	50.0%	-	51.9%	37.6%	66.0%	€ 2.0	€ 1.4	€ 2.5	[26]
*v*	Anogenital warts (women)	€ 41.3	-	90.0%	90.0%	-	-	€ 37.1	€ 37.1	€ 37.1	[19]
*z*	Anogenital warts (men)	€ 37.6	-	90.0%	90.0%	-	-	€ 33.8	€ 33.8	€ 33.8	[19]
*y*	RRP ‡	€ 45.5	-	85.0%	85.0%	85.0%	100.0%	€ 38.6	€ 38.6	€ 45.5	[7], [8]
*w*	**Total non-cervical conditions (q + r + s + t + u + v + z + y)**	**€ 381.7**						**€ 186.3**	**€ 176.9**	**€ 200.0**	
*w*	**Total burden (p + w)**	**€ 528.6**						**€ 291.0**	**€ 274.5**	**€ 315.7**	

‡ Respiratory recurrent papillomatosis (RRP).

Italian data not available. The mean prevalence rates reported by the reference were informed by the outcomes of an ongoing metanalysis on global data compiled by he Unit of Infections and Cancer at the Istitut Catala d'Oncologia. No confidence intervals for the reported mean values were given.

To ensure the inclusion of all relevant studies, we consulted the websites of official institutions, such as the Cochrane Gynaecological Cancer Group Trials Register, Global Health, and Clinical Trials.Gov. We also searched the grey literature for information from other relevant sources including the Italian Ministry of Health, the Italian National Institute of Health (NIH), the World Health Organization (WHO), the International Agency for Research on Cancer (IARC), the Catalan Institute of Oncology, and various Italian scientific societies [e.g., the Italian Society of Health Technology Assessment (SIHTA) and the Italian Society of Hygiene (SITI)], as well as sites dedicated to the rapid dissemination of scientific publications in the socioeconomic and healthcare fields [e.g., the Social Science Research Network (SSRN) and Research Papers in economics, (RePEc)]. Generic academic search engines (e.g., Google Scholar) and meta-engines (e.g., MetaCrawler) were also used to make the search as comprehensive as possible. Peer-reviewed journals, meetings, congresses, and other internet sources were searched up to December 2011 for presentations or communications on any additional data related to our research topic.

**Figure 2:**
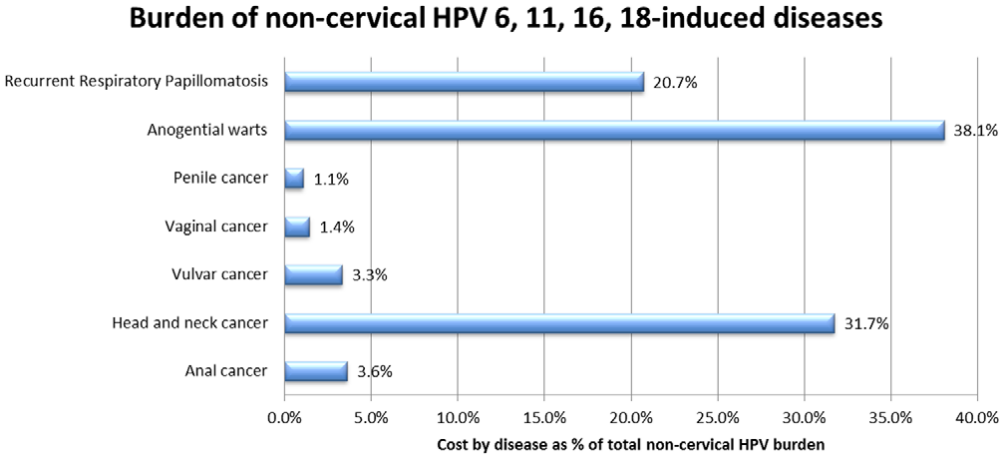
Burden of noncervical HPV 6, 11, 16, and 18-related diseases. The dominant prevalence of HPV 6 and 11 in anogenital warts and RRP, which were associated cumulatively with 56.9% of the total costs of noncervical HPV 6, 11, 16, and 18-related diseases, meant that the fraction of total costs for noncervical diseases attributable to HPV 6 and 11 was at least as large as the fraction of direct costs attributable to HPV 16 and 18.

All the studies used in the qualitative synthesis were required to have met at least one of the

**Figure 3:**
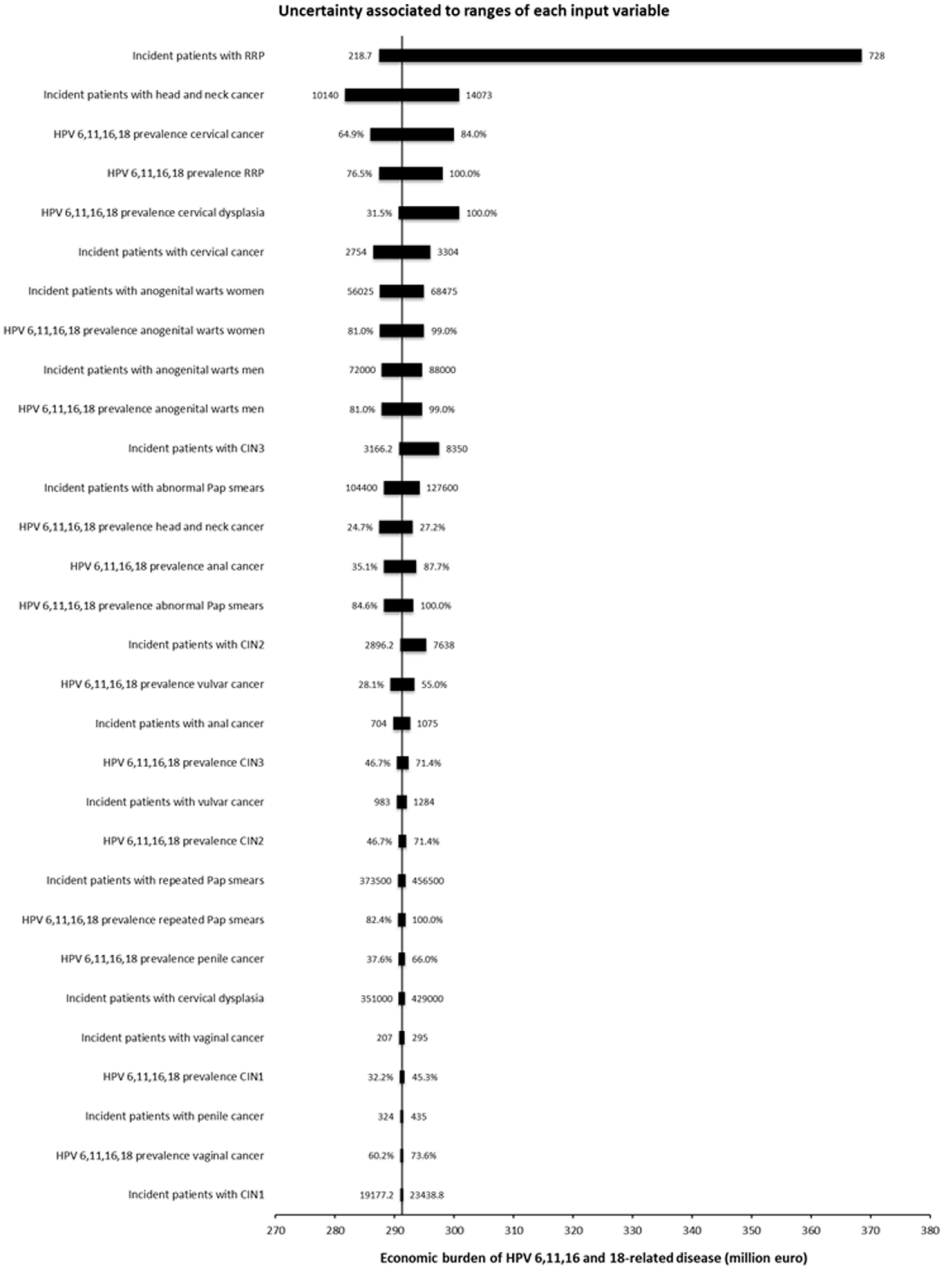
Sensitivity analysis. The Tornado chart reported in Figure 3 shows the changes in our valuation of the economic burden of HPV 6,11,16 and 18-related disease associated with ranges of values for each input variable. Uncertainty is mostly is associated with the widest swing in the incidence rate of RRP patients per year.

following inclusion criteria:

1. they report epidemiological data (incident cases by disease and prevalence of HPV) derived from population databases, such as national surveys or registries;
2. they report direct cost data accounted from the perspective of the payer, and expressed in monetary values for hospital Disease-Related Groups (DRGs) and outpatient tariffs. The DRG system aggregates all activities, including surgical interventions, drugs administered, materials, and personnel, for each individual diagnosis and stipulates the reimbursement tariff, which corresponds to the sum of all interventions provided, to be paid to the hospital. Outpatient medical treatments and interventions are stipulated in a similar way and reimbursed to the local territorial healthcare service. DRGs and tariffs represent the actual direct cost to the National Health Service and correspond to a codified hospital admission or outpatient intervention;
3. or they report an observational study of epidemiological data on HPV-induced diseases and their associated direct costs, observed simultaneously in the same cohort of patients.

Studies used in the quantitative estimates for the burden of diseases were additionally required to have met the following inclusion criteria:

1. they report Italian data relevant to the HPV-related diseases included in the cost analysis;
2. they report estimates of discounted lifetime cost per case.

#### Screening and data extraction

Two independent researchers screened the titles and abstracts of all the identified sources. The full-text versions were then obtained of all articles not excluded on the basis of the foregoing criteria. The best available data to inform the cost analysis were selected on the basis of consensus among the authors. In all phases of the literature review, disagreements were resolved by discussion among the entire team to reach a consensus.

**Table 5:** Fraction of HPV 6, 11, 16, 18 costs attributable to men.

	*Conditions*	*HPV 6,11,16,18 costs (mill Euro)*	*Lower bound*	*Upper bound*	*Percentage prevalence in men*	*HPV 6,11,16,18 costs attributable to men (mill Euro)*	*Lower bound*	*Upper bound*	*References*
		*a*	*b*	*c*	*d*	*a × d*	*b × d*	*c × d*	
*e*	Anal cancer	€ 6.7	€ 3.7	€ 9.2	40%	€ 2.7	€ 1.5	€ 3.7	[26]
*f*	Head and neck cancer	€ 59.1	€ 55.3	€ 60.9	80%	€ 47.3	€ 44.3	€ 48.8	[26]
*g*	Penile cancer	€ 2.0	€ 1.4	€ 2.5	100%	€ 2.0	€ 1.4	€ 2.5	[26]
*h*	Anogenital warts (men)	€ 33.8	€ 33.8	€ 33.8	100%	€ 33.8	€ 33.8	€ 33.8	[19]
*i*	RRP ‡	€ 38.6	€ 38.6	€ 45.5	70%	€ 27.0	€ 27.0	€ 31.8	[7], [8]
*l*	**Total costs attributable to men (e + f + g + h + i)**	**€ 183.7**	**€ 176.3**	**€ 200.1**		**€ 112.9**	**€ 108.0**	**€120.6**	
	% of non-cervical HPV 6, 11, 16, 18 burden					60.6%	58.0%	64.7%	
	% of total HPV 6, 11, 16, 18 burden					38.8%	37.1%	41.4%	

‡ Respiratory recurrent papillomatosis (RRP).

#### Assessment of the risk of bias

For the observational studies, the risk of bias was assessed by two independent researchers through the application of a checklist of essential items, which is reported in Table S1. We used an Excel spreadsheet, reported in Table S2, to estimate a summary risk of bias that considered three major criteria (methods of selecting study participants, methods of measuring exposure and outcome variables, and methods to control confounding), and two minor criteria (design-specific sources of bias, and statistical methods). Disagreements were resolved by discussion among the entire team to reach a consensus.

### Lifetime cost per case (Table 1)

We adopted an incidence-based approach, wherein a lifetime cost per case associated with each condition was applied to the estimated number of incident cases attributable to HPV 6, 11, 16, and 18 that occurred in men and women over a one-year period. We sought to develop cost-per-case estimates that represented the present value of the total direct medical costs that accrued from the time of diagnosis to follow-up. Whenever possible, we sought published estimates of discounted lifetime cost per case which were available for cervical cancer, cervical dysplasia, and anogenital warts. However, for other conditions, due to the scarcity of available Italian studies due to the paucity of available Italian studies, especially with respect to noncervical cancers and RRP, we estimated the discounted lifetime cost per case by modelling the costs related to diagnosis, treatment, and follow-up. Treatment paradigms for noncervical cancers (anal, head and neck, vaginal, vulvar, and penile) were based on the recommendations of generally accepted Italian Medical guidelines [16]–[18] and were reviewed by an independent Italian Medical Oncologist. Treatment patterns and associated direct lifetime costs for genital warts in Italy were modelled on the basis of an observational, prospective cost study conducted in 28 centres in Italy, in which 189 men and 142 women were enrolled [19]. In the absence of any available Italian data, we based the modelling of the treatment and follow-up of RRP on data from the US [20], [21]. Details of the assumptions made in the estimates of discounted lifetime cost per case are reported in the “Results” section.

Only direct medical costs from the perspective of the Italian National Health Service were included in the analysis. Costs were calculated on a DRG basis at 2006 tariffs [22]. However, given that a large variation in terms of tariff per DRG reimbursed could be observed across several Italian Regions, a mean national value was calculated for each DRG, weighted for the 2011 resident population. Outpatient costs were based on the 2006 national tariffs for specialists’ office consultations and procedures [23].

Incidence was estimated on the basis of the population resident in Italy in 2011, i.e., 31,213,168 women and 29,413,274 men, giving a total of 60,626,442. Census data on the Italian resident population were obtained from the Italian National Institute of Statistics [24].

Costs were adjusted for inflation at 2011 values using compound annual Italian National Consumer Price Indexes (NIC) [24] from 2005 (112.9%) or 2006 (110.6%).

Future costs (more than 12 months after diagnosis) that were associated with the follow-up of HPV-related diseases were not adjusted for inflation to match the nominal discount rate used (3%) to obtain their net present value. The chosen discount rate reflects the opportunity cost of financing from the perspective of a public payer (the Italian National Health Service in the present cost analysis) and it does not include the expected rate of inflation.

### Total direct costs by disease (Table 2)

Similarly to a recently published estimation of the burden of HPV-related disease in Europe [25], crude incident rates per 100,000 residents [26] were applied to the Italian resident population in 2011 [24] in order to estimate the total annual number of new cases by type of cancer in Italy:
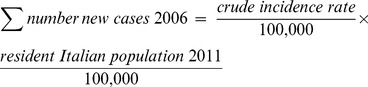



Crude incidence rates were calculated directly by source [26] from the accumulated number of cases observed in 22 Italian regional registries during the period 1998–2002.

The annual numbers of cases of abnormal Papanicolau (Pap) smears and colposcopies [15], cervical dysplasia [12], and anogenital warts [13], [27] were derived directly from the literature. Due to the lack of Italian data, the annual incidence of RRP was estimated on the basis of epidemiological data from the US [7], [8].

### Economic burden of cervical and noncervical HPV-related disease (Table 3 and Table s4)

To estimate the annual number of incident cases attributable to HPV 6, 11, 16, and 18, prevalence rates for HPV were identified from the literature review. For all cancer diagnoses (cervical, anal, head and neck, vulvar, and penile), the rate chosen was the pooled prevalence for HPV 6, 11, 16, and 18 [26]. Crude incidence rates (rates per 100,000 men/women per year) were obtained directly from source [26], and were based on microscopically -verified cases collected from 22 Italian regional registries during the period 1998– 2002. No Italian data on the prevalence of HPV in vaginal cancer were available: the rate used was informed by the results of an ongoing meta-analysis of global data compiled by the Unit of Infections and Cancer at the Institut Catala d'Oncologia in Barcelona [26], although no confidence intervals were given. The literature review provided Italian data on the prevalence rates for HPV 6, 11, 16, and 18 in abnormal PAP smears and colposcopies [15], and in diagnoses of cervical dysplasia [28] and anogenital warts [19]. Italian data on the prevalence of HPV in RRP were not available, and they were replaced by US data obtained from the literature review [7], [8].

Table 3 reports the HPV prevalence rates for HPV that were chosen to inform the cost model, as well as other and similar rates identified from the literature review. Due to the heterogeneity of the prevalence rates reviewed and the number of diseases involved, no attempt was made to perform standardisation of prevalence rates, meta-analyses or adjustments for the pyramidal stratification of the population observed.

To develop an aggregate monetary measure of the burden of HPV-related diseases, the fraction of incident cases attributable to HPV 6, 11, 16, and 18 was applied to the total lifetime costs per year for each disease (Table 4). The estimates of direct medical costs were summed for the nine diseases evaluated in order to approximate the total economic burden of cervical and noncervical diseases attributable to HPV in Italy.

Further details of the estimation approach are provided in the disease-specific sections of the Results.

### Statistical analysis

Incidence rates for HPV-related cancers included were obtained from 22 Italian registries [26]. Upper and lower bounds for the number of incident cancer cases per year were calculated as the 95% confidence intervals of the mean crude rate per 100,000 residents. A check for normality was performed for each diagnosis: the sample data did not provide sufficient evidence for rejecting the hypothesis of a normal distribution. Given that the sample size was smaller than 30, t-distribution 95% confidence intervals were computed using the formula for small samples:
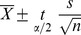



where: 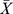 =  sample mean, t _α/2_  =  t-value with an area of α/2 to its right, s  =  sample standard deviation, n  =  sample size.

No upper and lower bounds were available for some cervical abnormalities [abnormal Pap smears, colposcopies, and diagnoses of cervical intra-epithelial neoplasia (CIN1 and CIN2)] or for anogenital warts. Due to the lack of Italian data, upper and lower bounds for annual incident cases of RRP were estimated from epidemiological data from the US [7], [8]. The use of the crude incidence rate did not consider the effect of a different population pyramid in between US and Italy compared to that of the US.

For most of the malignancies (including cervical, anal, head and neck, vaginal, and penile cancer) and cervical neoplasia (CIN1-3), the upper and lower bounds of the incidence of HPV 6, 11, 16, and 18 were based on 95% confidence intervals obtained directly from the literature [26]. No upper and lower bounds were available for vaginal cancer, the number of abnormal Pap tests, colposcopies, and diagnoses of cervical dysplasia attributable to HPV. Upper and lower bounds for the prevalence of HPV-related anogenital warts were identified from the literature [19]. Due to the lack of Italian data, the upper and lower bounds for the fraction of RRP attributable to HPV were estimated on the basis of annual incidence rates in the US [7], [8].

A sensitivity analysis of the impact of input uncertainty on the economic burden of HPV 6, 11, 16 and 18-related disease was performed using the SensIt’s Many Inputs, One Output option.

## Results

### Cervical Cancer

Cost estimates for cervical cancer were obtained from published Italian data from a retrospective observational study conducted for 351 patients [11]. This earlier study estimated the mean total direct costs for cervical cancer, which included those generated by the management of disease progression and recurrence. The observed costs reported in the reference [11] were not discounted, although the median duration of follow-up for the whole cohort was 23 months (range 1–89). We assumed that the mean costs per patient included in the study (n = 351) for the treatment of the primary tumour (€13,122) were incurred in year 1, whereas the mean costs associated with the management of disease progression and recurrence (€9,092) were incurred in year 2, and we discounted the latter by 3% (€8,827). The resultant discounted lifetime cost per case was estimated to be €24,276 at 2011 values.

Estimates of the annual incidence of cervical cancer in Italy were obtained from the literature [26]: a mean crude rate of 9.7045 per 100,000 women (95% C.I.: 8.824–10.585) was applied to the female population resident in Italy in 2006 in order to obtain a value of 2,933 incident cases per year (range 2,682–3,184). The mean number of incident cases per year fell within the range of published Italian data (2,262–4,000) [9], [10], [12], [29]. The mean prevalence rate of HPV 6, 11, 16 and 18 chosen to inform the cost model (72.1%) was within the 70 to 77% range identified from the literature review [30]–[33].

The fraction of the cost of cervical cancer in Italy that was attributable to HPV 6, 11, 16, and 18 was estimated to be €53.0 million (72.1% of the total direct costs for cervical cancer). When the upper and lower bounds for the prevalence of HPV 6, 11, 16, and 18 were considered, this cost ranged from €47.7 to €61.8 million.

### Cervical dysplasia

Although some regions in Italy started to implement screening programmes for cervical cancer in the 1970 s, an organised nationwide programme was only recommended in 1996, following the European Commission Guidelines on Quality Assurance in Cervical Cancer Screening. It is estimated that each year in Italy, approximately 5.2 million women aged 25–69 undergo opportunistic screening [12]. Data to inform the estimates of direct lifetime costs, the number of incident cases per year, and the prevalence rate of HPV 6, 11, 16, and 18 with respect to screening abnormalities, including the repeat of abnormal Pap tests and the management of Pap smears still positive after repetition, were obtained from published Italian data [15]. The annual cost associated with the management of abnormal Pap smears that were repeated after initial screening and were attributable to HPV 6, 11, 16, and 18 was estimated to be €6.3 million. The lifetime cost per case for the management of positive Pap smears that were abnormal after repetition was calculated over a three- year period. Women who still showed abnormal results after the repetition of the initial Pap test underwent a colposcopy with biopsy (€50 per procedure), a gynaecological examination (€21) and a repeat Pap test (€15). The same procedures would be repeated after 12 and 24 months, for a total discounted lifetime cost per case of €250 [15]. Of the total annual direct costs associated with the management of positive Pap smears, 93.8% were attributable to HPV 6, 11, 16, and 18 infections (€30.1 million). The management of positive Pap smears represented the strongest driver of direct costs associated with the management of cervical dysplasia, although upper and lower bounds could not be estimated. This finding was consistent with previously published Italian data [10], [15].

In our analysis, we estimated the total direct lifetime costs associated with the clinical and surgical management of cervical dysplasia from published Italian data [12]. Cost data were reported on a DRG basis using 2005 values. It was assumed that the excisional treatment for CIN, which included radiofrequency excision, cold-knife excision, and laser conisation, was performed in hospital, either as an outpatient or inpatient procedure. Disruptive treatments for CIN included laser vaporisation, cryotherapy, and diathermocoagulation. The cost of these treatments also included the cost of gynaecological examinations. A one year follow-up after negative colposcopy included the cost of two colposcopies with repeated Pap tests and gynaecological examinations.

Consistent with our primary methodological constraint, the estimated annual number of incident patients and cost data were derived from the same Italian source mentioned in the previous paragraph [12]. Upper and lower bounds for the number of annual incident cases were informed by data from the Italian Group for Cervical Cancer Screening (GISCi), which were obtained in a 2004 survey [34].

The cost of diagnosis of cervical dysplasia attributable to HPV 6, 11, 16, and 18 was derived from published data [28]. The mean prevalence rates of HPV 6, 11, 16, and 18 in Italy for CIN1 (38.1%), CIN2 (57.2%), and CIN3 (57.2%) and the relative confidence intervals were derived from regional cancer registries [26]. No comparable national Italian data were available for CIN1. A national Italian observational study reported prevalence rates for HPV 16 and, 18 in CIN2 and CIN3 lesions of 60.8% and 77.6% respectively [35]. Regional studies reported an incidence rate of HPV 16 and, 18 of more than 80% in all high- grade lesions (CIN2 and CIN3) [36], [37]. Data from regional registries might overestimate the prevalence of HPV due to the limited size of the population included.

The total cost associated with the management of cervical lesions, including the diagnosis and treatment of CIN1/2/3 stages was €34.4 million per year (range €34.4–€52.6 million). The fraction attributable to HPV 6, 11, 16, and 18 was €15.2 million (range €13.4–€17.5 million). A previous Italian cost analysis [10] estimated the total direct costs of low and high grade lesions to be in the range of €41.7 to €48.3 million. The difference could be explained mainly by the conservative estimate used in the source study of the number of new patients per year affected by cervical lesions, which was based on data from one regional Cancer Registry (Emilia-Romagna) [12]. Data from a national survey [34] provided a substantially higher estimate of the total incidence of CIN 2/3 and these data were used to inform the upper and lower bounds of the range. A sensitivity analysis using the two relevant sources of secondary data (the Emilia-Romagna Cancer Registry v. the GISCi data for 2004) was performed by Rossi et al. [12]. The most influential parameters were the number of cases of CIN 2/3, which increased the total cost of management of cervical dysplasia by 6.4% if the GISCi data were used. We chose the more conservative cost estimate to inform the base case cost analysis of the base case, because the GISCi survey found that the proportion of treated CIN lesions varied greatly among different centres [33].

### Non-cervical malignancies

We evaluated the direct costs associated with treatment and follow-up for HPV-related cases of five noncervical malignancies: cancer of the anus, head and neck, vulva, vagina, and penis. The diagnosis of head and neck cancer included oral, naso-pharyngeal, pharyngeal, and laryngeal cancers.

The lifetime cost per patient was modelled for each diagnosis on the basis of national treatment guidelines [16]–[18]. The main interventions that were included in the modelling of direct costs for the treatment phase of the management of noncervical malignancies were diagnosis, surgery, and radiotherapy. A 6-month follow-up included two clinical examinations conducted by a specialist and two two-sector computerised tomography (CT) scans. Weighted DRG and outpatient tariffs were attributed to each surgical procedure and medical treatment included. Treatment guidelines for malignancies indicate a maximum four-week period between diagnosis and surgery, radiotherapy or medical treatment [17]. Including a follow-up of 6 months, we assumed that the entirety of the management of patients with cancer would be conducted within a 12-month period; hence, cost data were not discounted. Although patients with cancer are usually monitored for a period longer than 6 months, no consistent data on long-term follow-up were available and, consequently, the relative costs were not included in the analysis. Similarly, no consensus was identified in the guidelines on the appropriateness of palliative chemotherapy, including the eligibility of patients by cancer stage, the choice of standard treatment, the dosage range, and the administration schedule. Although anecdotal evidence suggested that a significant proportion of cancer patients in Italy receive palliative chemotherapy after surgery and radiotherapy, the lack of controlled data prevented the estimate of its impact on the total costs of direct treatment. All the inputs that were chosen to inform the cost analysis were reviewed by an independent Medical Oncologist.

Crude rates of the annual incidence of noncervical cancers in Italy were obtained from the literature [26]. Due to the lack of information on crude incidence rates for different strata of the population, no attempt was made to adjust the annual incidence of cases to differences in the population.

The crude rates that we used to estimate the number of annual incident cases for head and neck cancers were confirmed by analogous rates collected from 1988 to 1992 by the Italian Istituto Superiore di Sanità (ISS) [38]. Table 3 reports the mean prevalence rates of HPV 6, 11, 16 and 18 chosen to inform the cost model and a range of prevalence rates identified from the review of the literature related to anal [39]–[46], vulvar [45]–[51], vaginal [51]–[52], head and neck [53]–[55] and penile cancer [56]–[58].

For all cancer diagnoses, most of the fraction that was attributable to HPV was constituted by types 16 and 18 (defined as high-risk or oncogenic), although a minor contribution of types 6 and 11 (defined as low-risk or nononcogenic) was observed in cervical and vaginal cancer. A small but significant prevalence (range 2 to 5%) of HPV 6 and 11 has been associated with noncervical cancers by an increasing number of authors [39], [45], [50], [53], [56]. However, it remains unclear in what proportion of these cases there is concomitant co-infection with a high-risk type and whether low-risk HPV types are “minority passengers” or the true cause of neoplastic lesions. With this caveat in mind, we decided to use the prevalence rates for the different types of HPV that were reported by the source publication [26] and to use pooled prevalence rates for HPV 6, 11, 16, and 18 to determine the fraction of the direct costs for each cancer that could be attributed to HPV. The prevalence rates that were chosen, which had been pooled in the source publication, should minimise the risk of overestimating the fraction of the direct costs of noncervical cancers attributable to an HPV infection.

The estimated fractions of the total annual direct costs attributable to HPV 6, 11, 16, and 18-induced malignancies are reported in Table 4.

Head and neck cancers were responsible for the highest annual burden of direct costs among the HPV 6, 11, 16, and 18-induced malignancies (€59.1 million, bounds €55.3–€60.9 million), followed by anal cancers (€6.7 million, bounds €3.7–€9.2 million), vulvar cancers (€6.2 million, bounds €4.2–€8.3 million), vaginal cancers (€2.7 million, bounds not available), and penile cancers (€2.0 million, bounds €1.4–€2.5 million).

### Anogenital warts

Lifetime direct costs per patient, stratified by gender and by diagnosis (new, recurrent, and resistant), were obtained from a published study [19]. The mean duration of follow-up was 3.56 (SD 3.36) months for men and 3.5 (SD 3.26) months for women; hence, costs were not discounted. The mean number of examinations was 2.5 (SD 3.3) per outpatient case. The mean number of examinations was considerably higher among women than men (4.6 vs. 0.9, respectively). The mean number of procedures was 1.8 (SD 1.8) per outpatient case. Forty-three patients were hospitalised. Of these, thirty nine (nine men, thirty women) were admitted to day hospital. The mean number of admissions among these patients was 1.2 (SD 0.4).

The estimated lifetime cost per incident case was calculated as the sum of the mean, inflation-adjusted direct costs incurred by new patients (€416 for women and €311 for men) and the probabilised, discounted, inflation-adjusted mean direct costs of a recurrent (€199 for women and €66 for men) and a resistant (€48 for women and €92 for men) episode observed one year after the initial diagnosis. Probability rates were assigned on the basis of the number of recurrent (50.6% for women and 34.8% for men) and resistant (28.2% for women and 38.5% for men) cases as a percentage of the annual number of newly diagnosed cases, assuming a steady state in the total number of genital warts treated in Italy [19]. To estimate the number of incident new cases per year in Italy, the observed rate of new patients among total cases (57.7% for women and 55.9% for men) on total cases [19] was applied to the estimated number of anogenital warts treated by gender by the Italian National Health Service each year [13], [27]. The HPV 6, 11, 16 and 18 prevalence rate chosen to inform the model (90%) fell in the range of prevalence rates (88 to 100%) identified from the review of the literature [59]–[60].

Consistent with the difference in the mean number of examinations observed between women and men in the present study, the estimated lifetime cost for the management of anogenital warts was significantly higher in women than in men (€663 vs. €470, respectively).

The total annual economic burden of anogenital warts in Italy that was attributable to HPV 6 and 11 amounted to €70.9 million, and was significantly higher for women (€37.1 million vs. €33.8 million, respectively). Data on upper and lower bounds were not available. Anogenital warts were the second highest contributor to the economic burden of HPV 6, 11, 16, and 18-related diseases in Italy, preceded by total cervical diseases (€104.6 million) and followed by head and neck cancers (€59.1).

### Respiratory Recurrent Papillomatosis (RRP)

No Italian data were available on the lifetime cost per patient, the annual number of incident cases, and HPV prevalence for this HPV 6 and 11-induced disease. Consequently, we proceeded to evaluate the economic burden of RRP using the relatively few data available in the international literature. Because of the lack of benchmarks for calibrating the inputs to the cost model, we decided to use a conservative approach to the valuation of the annual direct costs that were attributable to RRP.

To estimate the lifetime cost per patient, elective surgical procedures and medical treatments were selected on the basis of published reports from the US [20], [21]. Treatment assumptions included the management of complicated respiratory infection, three acute clinical treatments, 4.4 surgical procedures, and a tracheotomy rate of 11% per year [20]. Italian DRG tariffs for 2006 were applied to the selected procedures and adjusted for inflation to values for 2011. The duration of the disease was estimated to be 4.2 years [20], [21]. All costs incurred after the first 12 months of treatment were not increased for inflation and were discounted at a rate of 3% per year to obtain their net present value expressed in 2011 Euro.

To estimate the number of incident cases of RRP per year, we applied a crude incidence rate (0.4 per 100,000 residents) [7], [8] to the Italian population of 2011. Similar incidence rates for juvenile RRP of 0.35 per 100,000 residents per annum were recorded in Denmark (population 5.4 million) for the period 1974–-1999 [60]. The prevalence rate for HPV 6 and 11 that was chosen (85%) was informed by data in the published literature [7], [8], [61]–[62]. The estimated total lifetime incident cost for RRP in Italy that was attributable to HPV 6 and 11 was €38.6 million (range €38.6–€45.5 million). Because of the lack of Italian epidemiological data, the cost estimate for RRP that was used to inform the model for the total HPV economic burden was deliberately chosen from the lower bound of the expected cost range.

### Economic burden of cervical and noncervical HPV 6, 11, 16, and 18-related diseases

The total direct costs (expressed in 2011 Euro) associated with the annual incident cases of the nine HPV-related conditions included in the analysis were estimated to be €528.6 million, with a plausible range of €480.1– 686.2 million. The fraction attributable to HPV 6, 11, 16, and 18 was €291.0 (range €274.5–315.7 million), accounting for approximately 55% of the total annual burden of HPV-related disease in Italy.

Under the base case assumptions, the fraction of the annual direct costs of noncervical HPV-related diseases that could be attributed mainly to HPV 6 and 11 (which were almost exclusively responsible for anogenital warts [19] and RRP [7], [8]) corresponded to 58.8% of the total direct costs attributable to HPV 6, 11, 16, and 18-related diseases (Figure 2). These costs were mainly driven by the high number of incident cases of anogenital warts treated each year in Italy and the large fraction of discounted lifetime costs for RRP that could be attributed to HPV 6 and 11.

A sensitivity analysis was performed on the critical inputs used to inform the model, namely the number of incident cases per year and the HPV 6, 11, 16 and 18 prevalence for all the nine diseases included in the cost analysis. The Tornado chart reported in Figure 3 shows that the uncertainty in our valuation of the economic burden of HPV 6, 11, 16 and 18-related disease is mostly associated with the range of possible incidence rates of RRP patients per year.

We have also run a further scenario analysis, in which the discount rate was set at 2.5%, 3% (base case) and 3.5%; in this case, we obtain values for the economic burden of HPV 6,11,16 and 18-related diseases of €291.5, €291 (base case) and €290.4 million.

## Conclusions

To our knowledge, the present study is the first attempt to evaluate the annual economic burden of diseases associated with HPV 6, 11, 16, and 18 infections in Italy. From the aggregate measure of the cost estimates, we make three basic observations that could inform future public health choices in terms of programmes of immunisation and secondary prevention of HPV infections.

First, the dominant prevalence of HPV 6 and 11 in cases of anogenital warts and RRP meant that the total costs for noncervical diseases that were attributable to the low-risk HPV types 6 and 11 were at least as high as the direct costs attributable to the high-risk HPV types 16 and 18. This occurred because anogenital warts and RRP were associated cumulatively with 58.8% of the total costs of noncervical HPV 6, 11, 16, and 18-related diseases. This observation was consistent with data already in the literature from the US [4], [21], [59], notwithstanding all the differences in terms of access to healthcare and screening programmes and the substantially different costs of surgical procedures and medical treatments between Italy and the US. Regardless of the differentiation between “high” vs. “low” risk, or “oncogenic” vs. “nononcogenic” HPV types 6 and 11 might have the same importance as HPV 16 and 18 in terms of drivers of the economic burden of noncervical HPV-related disease. Figure 2 shows the costs attributable to each noncervical HPV 6, 11, 16, and 18-related disease expressed as a percentage of the total economic burden of noncervical pathologies.

Second, Table 5 shows that the economic burden attributable to noncervical HPV 6,11,16, and 18-related diseases was higher among men than among women (60.6% vs. 39.4% of the total, respectively). The economic burden among men represented more than one third (38.8%) of the total direct costs of HPV 6, 11, 16, and 18-related diseases, including cervical conditions (cervical cancer, dysplasia, and CIN1/2/3 lesions). This observation was consistent with previously published data [4], [59], and could inform Italian national and regional evaluations of the economic value of extending the anti-HPV immunisation programmes to cohorts of boys.

Third, RRP was the fourth largest driver of the economic burden of HPV 6, 11, 16, and 18-related diseases in Italy (€38.6 million; 13.3% of total costs in the base case). However, epidemiological and cost data are still not available for Italy. To reduce the uncertainty that is presently associated with the estimates of the economic burden of RRP, the incidence, prevalence, aetiopathogenesis, and management of RRP should be observed in Italy over a sufficient period of time.

The present study has several limitations that merit acknowledgement. Italian sources of cost data were limited and the quality of available information was variable. Published estimates of lifetime cost per case were only available for cervical cancer [11], cervical dysplasia [12], [15], and anogenital warts [19]. Cost information derived from primary data (retrospective observation of treated patients from diagnosis to follow-up and collection of actual cost data) was available only for cervical cancer [11] and anogenital warts [19], whereas the cost estimates for cervical dysplasia were obtained by applying DRG tariffs for surgical procedures and medical treatments recommended by Italian medical guidelines [12], [15].

The data used to derive the lifetime cost per patient for noncervical cancers were not comprehensive, but were based on the valuation of the interventions recommended by the generally accepted Italian Medical guidelines [16]–[18]. The derived cost estimates omitted the marginal economic impact of long-term complications and recurrences, palliative chemotherapy, and GP prescriptions. In these instances, it is likely that the cost estimates underestimated the actual lifetime cost of disease. These limitations should be considered in future research, but, in our opinion, do not undermine the validity of the cost estimates in view of their marginal impact on the total economic burden of HPV-related disease. As an example, we can estimate the economic impact of palliative chemotherapy, which is possibly the most expensive medical intervention among those not considered. Let us assume that half of the patients treated each year for noncervical, HPV-related cancers (14,309; range: 5,990–8,318) undergo three cycles of chemotherapy in the year following the initial diagnosis. The DRG 410 sets the cost of one cycle of chemotherapy delivered in day hospital at €465.2 (2011 inflation-adjusted value). Hence, the overall cost for three cycles discounted at a rate of 3% would be equal to €1,353.7 per patient. The estimated cost of palliative chemotherapy for patients with noncervical cancer would be €9.7 (range €8.1–€ 11.3) million per year, which represents less than 2% of the total economic burden of HPV-related diseases in Italy (€528.6 million per year).

The attribution of direct costs to HPV and HPV 6, 11, 16 and 18 was derived mostly from prevalence rates obtained from Italian regional registries. The use of population-based cancer registry data to assess the proportion of diseases attributable to HPV might have resulted in an overestimation of the contribution of HPV, due to its high prevalence in healthy subjects [21], [46].

Cost data based on DRG tariffs did not capture the actual direct costs incurred by hospitals and outpatient practices. Direct or indirect nonmedical costs were also not included, due to the lack of availability of published Italian data. One aim of any future research should be to fill some of the gaps in the Italian epidemiological and cost data to reduce the uncertainty associated with the present estimates.

In summary, our results have provided an approximate but plausible estimate of the significant economic burden imposed by the most prevalent HPV-related diseases on the Italian welfare system. The fraction of total direct lifetime costs attributable to HPV 6, 11, 16, and 18 infections and the economic burden of noncervical, HPV-related diseases carried by men were found to be cost drivers that are relevant to the making of informed decisions on future investments in programmes to prevent HPV infection.

## Supporting Information

Appendix S1 Details about the search strategy and the terms used to query the main following electronic databases: MEDLINE (PubMed), EMBASE (accessed through OVID SP), CDC, CINHAL, and NIHR HTA, covering the period 1990–2011.
(DOCX)

Table S1 Checklist of essential items used by two independent researchers to assess the susceptibility to bias of observational studies.
(DOCX)

Table S2 Risk assessment for major systematic biases of the studies identified by the systematic literature review which reported epidemiological or economic data used to inform the burden of disease.
(DOCX)
