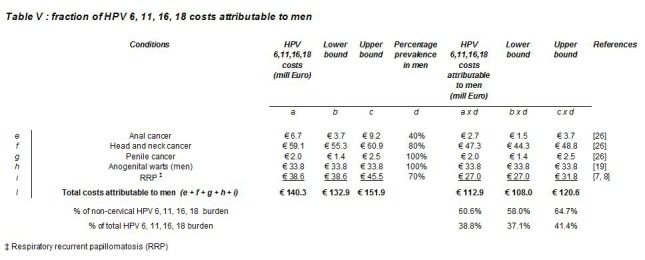# Correction: Economic Burden of Human Papillomavirus-Related Diseases in Italy

**DOI:** 10.1371/annotation/39ddb7a9-3e7e-4544-a226-2e2b872a5116

**Published:** 2013-09-27

**Authors:** Gianluca Baio, Alessandro Capone, Andrea Marcellusi, Francesco Saverio Mennini, Giampiero Favato

In Table 5, the Total costs attributable to men (e + f + g + h + i) for columns a, b, and c were incorrect. Please see the corrected Table 5 here: 

**Figure:**