# Supplementary material for: Economic Burden of Human Papillomavirus-Related Diseases in Italy
Source: PLoS One. 2012 Nov 21;7(11):e49699. doi: 10.1371/journal.pone.0049699 (PMC3504125; doi:10.1371/journal.pone.0049699)
Supplement: Appendix S1 — Details about the search strategy and the terms used to query the main following electronic databases: MEDLINE (PubMed), EMBASE (accessed through OVID SP), CDC, CINHAL, and NIHR HTA, covering the period 1990–2011. (DOCX) [file pone.0049699.s001.docx]

# Appendix S1.

# Search terms used in electronic databases

MEDLINE (Pubmed)

## Search 1

((((((((("uterine cervical neoplasms"[MeSH Terms] OR ("uterine"[All Fields] AND "cervical"[All Fields] AND "neoplasms"[All Fields]) OR "uterine cervical neoplasms"[All Fields] OR ("cervical"[All Fields] AND "cancer"[All Fields]) OR "cervical cancer"[All Fields]) OR ("uterine cervical dysplasia"[MeSH Terms] OR ("uterine"[All Fields] AND "cervical"[All Fields] AND "dysplasia"[All Fields]) OR "uterine cervical dysplasia"[All Fields] OR ("cervical"[All Fields] AND "dysplasia"[All Fields]) OR "cervical dysplasia"[All Fields] OR "cervical intraepithelial neoplasia"[MeSH Terms] OR ("cervical"[All Fields] AND "intraepithelial"[All Fields] AND "neoplasia"[All Fields]) OR "cervical intraepithelial neoplasia"[All Fields] OR ("cervical"[All Fields] AND "dysplasia"[All Fields]))) OR ("vulvar neoplasms"[MeSH Terms] OR ("vulvar"[All Fields] AND "neoplasms"[All Fields]) OR "vulvar neoplasms"[All Fields] OR ("vulvar"[All Fields] AND "cancer"[All Fields]) OR "vulvar cancer"[All Fields])) OR ("vaginal neoplasms"[MeSH Terms] OR ("vaginal"[All Fields] AND "neoplasms"[All Fields]) OR "vaginal neoplasms"[All Fields] OR ("vaginal"[All Fields] AND "cancer"[All Fields]) OR "vaginal cancer"[All Fields])) OR ("anus neoplasms"[MeSH Terms] OR ("anus"[All Fields] AND "neoplasms"[All Fields]) OR "anus neoplasms"[All Fields] OR ("anal"[All Fields] AND "cancer"[All Fields]) OR "anal cancer"[All Fields])) OR ("pharyngeal neoplasms"[MeSH Terms] OR ("pharyngeal"[All Fields] AND "neoplasms"[All Fields]) OR "pharyngeal neoplasms"[All Fields] OR ("pharynx"[All Fields] AND "cancer"[All Fields]) OR "pharynx cancer"[All Fields])) OR ("nasopharyngeal neoplasms"[MeSH Terms] OR ("nasopharyngeal"[All Fields] AND "neoplasms"[All Fields]) OR "nasopharyngeal neoplasms"[All Fields] OR ("nasopharynx"[All Fields] AND "cancer"[All Fields]) OR "nasopharynx cancer"[All Fields])) OR ("condylomata acuminata"[MeSH Terms] OR ("condylomata"[All Fields] AND "acuminata"[All Fields]) OR "condylomata acuminata"[All Fields] OR ("anogenital"[All Fields] AND "warts"[All Fields]) OR "anogenital warts"[All Fields])) OR ("Recurrent respiratory papillomatosis"[Supplementary Concept] OR "Recurrent respiratory papillomatosis"[All Fields] OR "recurrent respiratory papillomatosis"[All Fields])) OR (juvenile[All Fields] AND recurrent[All Fields] AND ("papilloma"[MeSH Terms] OR "papilloma"[All Fields] OR "papillomatosis"

[All Fields])) AND (hasabstract[text] AND ("1990/01/01"[PDAT] : "2011/12/31"[PDAT]))

Result: 67,103 records

## Search 2

HPV[All Fields] AND ("italy"[MeSH Terms] OR "italy"[All Fields]) AND (hasabstract[text] AND ("1990/01/01"[PDAT] : "2011/12/31"[PDAT]))

Result: 864 records

## Search 1 and 2

(#1) AND #2 AND (hasabstract[text] AND ("1990/01/01"[PDAT] : "2011/12/31"[PDAT]))

Result: 503 records

EMBASE (accessed via OVID SP)

## Search 1

(cervical cancer or cervical dysplasia or vulvar cancer or vaginal cancer or anal cancer or penile cancer or (head and neck cancer) or oral cavity cancer or pharynx cancer or nasopharynx cancer or anogenital warts or recurrent respiratory papillomatosis or juvenile recurrent respiratory papillomatosis).af.

limit 1 to yr="1990 – 2011

Result: 58,937 records

## Search 2

(HPV and Italy).af

limit 2 to yr="1990 –- 2011"

Result: 1,821 records

## Search 1 and 2

1 and 2

Result: 586 records

ALL OTHER DATABASES

Keywords searched:

HPV and Italy (limit to year “1990 – 2011”)
