# Supplementary material for: Economic Burden of Human Papillomavirus-Related Diseases in Italy
Source: PLoS One. 2012 Nov 21;7(11):e49699. doi: 10.1371/journal.pone.0049699 (PMC3504125; doi:10.1371/journal.pone.0049699)
Supplement: Table S1 — Checklist of essential items used by two independent researchers to assess the susceptibility to bias of observational studies. (DOCX) [file pone.0049699.s002.docx]

# Table S2.

# Tool for assessing susceptibility to bias in observational studies
