# Supplementary material for: Economic Burden of Human Papillomavirus-Related Diseases in Italy
Source: PLoS One. 2012 Nov 21;7(11):e49699. doi: 10.1371/journal.pone.0049699 (PMC3504125; doi:10.1371/journal.pone.0049699)
Supplement: Table S2 — Risk assessment for major systematic biases of the studies identified by the systematic literature review which reported epidemiological or economic data used to inform the burden of disease. (DOCX) [file pone.0049699.s003.docx]

# Table S3.

# Tool for assessing susceptibility to bias in observational studies
